# Immunological mechanisms of exercise therapy in dyslipidemia

**DOI:** 10.3389/fphys.2022.903713

**Published:** 2022-08-08

**Authors:** Karsten Krüger, Paulos Tirekoglou, Christopher Weyh

**Affiliations:** Department of Exercise Physiology and Sports Therapy, Institute of Sport Science, Justus-Liebig-University Giessen, Giessen, Germany

**Keywords:** inflammation, immunosenescence, sports therapy, metabolism, cardiovascular disease

## Abstract

Numerous studies demonstrated the strong link between dyslipidemia and the cardiovascular risk. Physical activity and exercise represent effective prevention and therapy strategies for dyslipidemia and at the same time counteract numerous comorbidities that often accompany the disease. The physiological mechanisms are manifold, and primary mechanisms might be an increased energy consumption and associated adaptations of the substrate metabolism. Recent studies showed that there are bidirectional interactions between dyslipidemia and the immune system. Thus, abnormal blood lipids may favor pro-inflammatory processes, and at the same time inflammatory processes may also promote dyslipidemia. Physical activity has been shown to affect numerous immunological processes and has primarily anti-inflammatory effects. These are manifested by altered leukocyte subtypes, cytokine patterns, stress protein expression, and by reducing hallmarks of immunosenescence. The aim of this review is to describe the effects of exercise on the treatment dyslipidemia and to discuss possible immunological mechanisms against the background of the current literature.

## Introduction

One of the key risk factors for the development of cardiovascular disease is hyper- and dyslipidemia. Both terms denote lipid metabolism disorders which are not quite uniformly defined, as in some cases the transported blood lipids, individual lipoprotein fractions or a combination of blood lipids and lipoproteins are used for clinical diagnosis. Hyperlipidemia mainly describes hypercholesterolemia (cholesterol >200 mg/dl or 5.2 mmol/L), hypertriglyceridemia (elevated triglycerides >150 mg/dl or 1.7 mmol/L) and combined hyperlipidemia (elevated cholesterol and elevated triglycerides). Altered lipoproteins mean hyperlipoproteinemia (mostly increased LDL), hypolipoproteinemia (mostly decreased HDL), and dyslipoproteinemia (high LDL and low HDL levels). Hyperlipidemia is suggested to be a major risk factor for the development of atherosclerosis. In particular, an increase in LDL is associated with an increased risk of cardiovascular disease, including coronary heart disease (CHD) and stroke (Pressler, 2017).

## Lipid metabolism disorders as a risk factor for cardiovascular diseases

Cholesterol-rich LDL and other apolipoprotein B (ApoB)-containing lipoproteins, including very low-density lipoproteins (VLDL), intermediate density lipoproteins (IDL) and lipoprotein(a), m[Lp(a)], have a direct impact on the development of atherosclerosis and its cardiovascular consequences. The increased concentrations of these lipid fractions in the blood lead to functional changes in endothelial barrier function because lipids travel freely between the vessel lumen and the vessel wall. Hence, LDL and other ApoB-containing lipoproteins enter and leave the arterial intima especially at sites vulnerable for plaque formation. This subsequently leads to a retention and accumulation of cholesterol-rich lipoproteins within the intima. It is suggested that the higher the LDL-C concentrations, the higher the probability of retention, what creates the basis for the development of an arteriosclerotic plaques (Goldstein and Brown, 2015). In endothelial cells, enzymatic systems are activated, which induce an overproduction of reactive oxygen species (ROS). The increased oxidative stress in turn exhibits pro-atherogenic effects and induces pro-inflammatory signal cascades. The activated endothelium starts to express various inflammatory cytokines, chemokines and to express adhesion molecules, causing leukocytes adherence to the vascular endothelium. Depending on the degree of activation, immune cells transmigrate and infiltrate the vessel wall. Increased ROS formation and inflammation inhibits the production and bioavailability of nitric oxide (NO), which further exacerbates endothelial dysfunction (van Diepen et al., 2013).

## Inflammatory processes interact with dyslipidemia

Migrating monocytes play a special role in these processes, because these cells differentiate into macrophages and transform into foam cells after taking up modified LDL *via* scavenger receptors (Sorci-Thomas and Thomas, 2016). Macrophage foam cells (MFCs) play a crucial role in the initiation and progression of atherosclerosis. At the same time, LDL cholesterol directly contributes to activation of the NLRP3 inflammasome, a cytosolic multiprotein complex of the innate immune system, which enhances the endothelial inflammatory response (Grebe and Latz, 2013). The multiple local inflammatory activities lead to an increased systemic production of C-reactive protein (CRP), tumour necrosis factor-alpha (TNF-α), interleukin (IL)-6 and IL-1, which are also thought to play a causal role in the development and progression of atherosclerosis. Conversely, systemic inflammatory processes, which are promoted by obesity, nicotine abuse, psychological stress or various autoimmune diseases, can promote local endothelial dysfunctions (Alack et al., 2019).

The subclinical inflammatory process is bi-directionally linked to lipid metabolism. On the one hand, inflammatory activities lead to increased cholesterol reverse transport and thus to increased formation of VLDLs (Khovidhunkit et al., 2004). On the other hand, circulating LDL has an increased tendency to oxidize, which explains the increased plasma levels of oxidized LDL (oxLDL) in patients with chronic inflammatory diseases (García-Gómez et al., 2014). OxLDL has a strong atherogenic effect, can promote inflammatory processes at the endothelium and even have a toxic effect on endothelial cells (Roma et al., 1992). Conversely, many immune cells interact with different classes of lipids and thus control their differentiation. In particular, the development of inflammatory leukocyte subtypes is favored (Hubler and Kennedy, 2016).

## Immune ageing as a driver of atherosclerotic processes

Physiological aging is frequently accompanied by a progressive increase in the concentration of circulating inflammatory cytokines. In particular, T cell senescence appears to have a bidirectional relationship with the development of chronic low-grade inflammation (Franceschi, 2007). On the one hand, chronic inflammation is a driver of cellular senescence by constantly activating immune cells. On the other hand, senescent cell types often present a more pro-inflammatory phenotype and thus become a potent source for the secretion of pro-inflammatory cytokines (Davalos et al., 2010).

While the described immune aging processes are part of the physiological remodeling of the immune system in old age, they are accelerated by lifestyle factors, such as overnutrition, inactivity and the resulting overweight. Dyslipidemia may also accelerate the immune aging process. Data suggests that the metabolism of immune cells, especially T cells, is influenced by the altered lipid concentration in the environment. In particular, cholesterol metabolism of T cells is disturbed which was demonstrated to inhibit their proliferation and favor the development of pro-inflammatory phenotypes (Boßlau et al., 2021; Kim et al., 2021). Progressive “inflammaging”, which is bi-directionally accelerated by lipid changes, leads to a vicious circle of dyslipidemia and maladaptive immune-aging. Inflammatory signaling pathways are continuously activated and initiate atypical cytotoxic activity toward endogenous structures such as the endothelium, which may ultimately favor the development of cardiovascular diseases (CVDs) (Nakajima et al., 2002).

Typical inflammatory markers, produced by many types of senescent cell in atherogenesis, are IL-1a, IL-1β, IL- 6, IL-8, IL-18 and TNF-a (Coppé et al., 2010; Freund et al., 2010; Prattichizzo et al., 2016). These cytokines promote inflammation locally in a paracrine manner and perhaps at a systemic level (Stojanović et al., 2020). The chronic inflamed endothelium tends to become dysfunctional and allow deposits to build up, which also recruit adaptive immune cells by way of their inflammatory process (Libby et al., 2002). Besides MFCs, T cells play a curricular role as major regulators of atherogenesis. They can either act as positive or negative modulators of plaques. In particular, CD4^+^ cells are considered to be an important cell type (Daugherty and Rateri, 2002; Hansson, 2005). Activated effector memory (EM) and central memory (CM) subtypes accumulate and thus stimulate the progression of atherogenesis. Inside the atherosclerotic plaques the cells release pro-inflammatory cytokines and bind to antigens of cholesterol-rich lipoproteins (Ammirati et al., 2012). After feeding a hypercholesterolemia-inducing diet, antigen-specific T cell clones actively expand in the plaque (Centa et al., 2018). CD4^+^ cells in plaque are specific for oxLDL since LDL is a relevant autoantigen that could drive the autoimmune response against intrinsic proteins in the atherosclerotic plaque (Stemme et al., 1995) (Wolf and Ley, 2019).

These findings are in agreement with our own data demonstrating that T effector memory re-expressing CD45RA (T-EMRA cells) are highly associated with body fat (Boßlau et al., 2021). T-EMRA cells are also strong producers of inflammatory cytokines and exhibit cytotoxic activity toward the endothelium, probably contributing to plaque erosion. Therefore, they tend to be associated with unstable plaques and with severe CVD, and are considered a predictor of mortality in the elderly (Stojanović et al., 2020). In addition to the pro-atherogenic properties of highly differentiated CD4^+^ T cells, also CD8^+^ cells are involved in atherosclerotic remodeling, although little is known about their role in pathogenesis (Carrasco et al., 2022). It is suggested that CD8^+^ cells contribute to inflammatory processes in the plaque, which might favor its instability. However, the antigen specificity of these CD8 lymphocytes is poorly understood (Kolbus et al., 2010; Kyaw et al., 2013).

## Interaction of stress proteins with dyslipidemia

Heat shock proteins (HSPs) are expressed when cells are exposed to cellular stress factors such as hypoxia or infection. In addition, there are data showing that HSPs are also increased intracellularly and extracellularly in endothelial cells during atherosclerosis, and increased extracellular levels are associated with systemic inflammation (Xu et al., 2012). It is well known that proteins like HSP 70 can modulate the inflammatory response in the context of cellular stress reactions (Noble and Shen, 2012). Already in early stages of atherosclerosis, an increased expression and release of HSPs was shown. It is suggested that the HSP-induction results from one or a combination of factors, such as hyperlipidaemia, diabetes, smoking, and hypertension (Zhu et al., 2003)**.** Increased oxidative stress might be the primary trigger which leads to the induction of HSP expression in vascular smooth muscle cells and in serum (Liao et al., 2000). It was further demonstrated that HSP60 serum levels correlate with the total cholesterol, LDL, and ApoB and negatively with adiponectin, and the intensity of HSP expression also correlates positively with the severity of atherosclerosis (Pockley, 2002). Another source of HSP60 are endothelial cells which are stressed by dyslipidemia. HSP60 synthesis and release modifies endothelial cells to targets of HSP60 specific T cells, as they express increased adhesion molecules. This favors the formation of macrophage-derived foam cells (Hashikawa et al., 2021). Mechanistically, HSP60 has been shown to contribute directly to the development of arteriosclerosis due to an increased synthesis of E-selectin andVCAM-1 within the endothelial cell (Kol et al., 1999). In response of chronic upregulation, these adhesion molecules participate in monocyte accumulation in the arterial intima. These results are supported by studies indicating that HSP 60 is selectively located in atherosclerotic lesions rather than non-atherosclerotic areas of the arterial wall (Guisasola et al., 2009).

The physiological processes triggered by increased HSP expression inside and outside the cell are divergent. While HSPs exhibit protective and anti-inflammatory activity intracellularly, increased extracellular levels results in pro-inflammatory signals (De et al., 2000). This divergence appears to be based in the fact that increased extracellular appearance of HSPs is often the result of a chronic and marked HSP expression, which is a consequence of cardiovascular stress, such as chronic exposure to oxidative stress. Then, the protective function is partially lost, and HSPs may promote atherosclerosis (Krüger et al., 2019).

## Treatment of lipid levels is a key to risk reduction

The clear links between lipid metabolism disorders and cardiovascular morbidity and mortality already make it clear that there is, conversely, also a causal relationship between a therapeutically reduced LDL level, increased HDL and a reduction in the cardiovascular risk profile. Scientifically, this is undisputed, so that the treatment of elevated lipid levels aimed at individual targets represents a key component of risk modification in the primary and secondary prevention of cardiovascular disease (Pressler, 2017). The steadily increasing prevalence of dyslipidemia requires the progressive use of lifestyle measures in addition to drug therapy. Many randomized controlled trials and meta-analyses have convincingly demonstrated that regular physical activity is effective in both the prevention and treatment of hyperlipidemia and dyslipidemia. Conversely, this is underpinned by the fact that a sedentary lifestyle is a major cause of dyslipidemia and cardiovascular disease (Albarrati et al., 2018).

## Exercise therapy and sports for dyslipidemia

Epidemiological studies and prospective intervention studies provide clear evidence that physical activity (in the sense of total activity) and also exercise training can reduce cardiovascular morbidity and mortality by improving lipid profiles (Rhee et al., 2019). The best relationship can be established between the activity level and increased HDL values as well as reduced triglyceride values. Regarding HDL levels, a meta-analysis with 19 included studies showed that HDL2-C levels increased significantly through regular endurance training. It was interesting to note that this effect also occurred independently of changes in body weight and BMI (Kelley and Kelley, 2006). There are contradictory data regarding the effect of physical activity on LDL and its subfractions. However, with regard to the small LDL particles, which are classified as particularly atherogenic, there are indications that these are reduced by physical training (Varady et al., 2005).

The mechanisms of these effects are manifold. The primary one is certainly the metabolic effect of exercise, as the increased energy metabolism of active people increases both the metabolism due to more muscular activity and, in the long term, the resting metabolism. Accordingly, an increased calorie intake can be at least partially compensated for by exercise. Due to the increased energy turnover and the addressed substrate utilization pathways, different systems of the body adapt functionally and structurally to the corresponding metabolic challenge. For example, the reduction in plasma triglyceride concentration is primarily due to the upregulation of lipoprotein lipase (LPL) activity and quantity in skeletal muscle. Moderate endurance exercise in particular uses intramuscular triglycerides as a primary source of energy, depending on the duration (Watt et al., 2002). In addition to enzyme systems of the musculature, other processes of lipid metabolism are also promoted by physical activity. For example, exercise increases the activity of hormone-sensitive lipases (HSLs) in adipose tissue and muscle, allowing triglycerides to be more efficiently converted to free fatty acids and mobilized from the tissues. The expression of plasma membrane fatty acid binding proteins, such as FABPPM, also increases with regular activity, allowing fatty acids to enter the muscle cell more efficiently. The intramuscular capacity to bind free fatty acids in the cytosol and transport them to the mitochondria for β-oxidation is also improved by regular activity (Ringseis et al., 2011). The mechanism of HDL increase through exercise is only partially understood. One cause seems to be the increase in adiponectin secretion through exercise. Adiponectin is a protein hormone that is predominantly produced by adipocytes and is involved in the regulation of glucose levels and fatty acid breakdown. Adiponectin concentrations could be positively correlated with HDL-C levels in men and women in several studies. Thus, as an anti-inflammatory adipokine, adiponectin could also have a metabolic mediator role, since an influence of adipokine on HDL synthesis in the liver has been shown (Greene et al., 2012). Furthermore, regular activity reduces hepatic triglyceride secretion. Since peripheral HDL levels increase at the same time, both processes could be related in the context of activity (Couillard et al., 2001).

## Immune-regulating effects of exercise on dyslipidemia

In addition to affecting blood lipids, physical exercise has a positive immune-regulating effect, which can foster anti-inflammation and thus can promote metabolic and cardiovascular health. Two mechanisms are important here. Exercise seems to positively influence cellular immune senescence, in addition to the release of pro- and anti-inflammatory cytokines. Thus, exercise affects both systems, which, as described above, are in a bidirectional relationship with chronic low-grade inflammation (Rosa-Neto et al., 2022).

### Exercise affects T cell aging

With regard to T cell differentiation and expression of pro-inflammatory subtypes, research shows that training status and the starting of exercise training are related to senescent hallmarks (Duggal et al., 2019; Weyh et al., 2020). Cross-sectional data show that trained individuals or those with a long history of exercise have fewer senescent CD4^+^ as well as CD8^+^ CD28^−^CD57^+^ cells. Additionally, they had fewer differentiated CD4^+^ CM cells, CD8^+^ CM and EM cells and fewer highly differentiated CD4^+^ and CD8^+^ T-EMRA cells (Spielmann et al., 2011; Minuzzi et al., 2018). As described above, these cell types also play an important role in the development of atherosclerosis and plaque formation, in addition to being associated with antigens of cholesterol-rich lipoproteins. In parallel, a higher proportion of naïve T cells is observed. The beginning of regular exercise in previously inactive subjects can also affect hallmarks of T-cell aging. After only 3 weeks of endurance training in prediabetic subjects, a proportional increase in naïve and central memory T cells was found, while at the same time the proportion of senescent CD8^+^ T-EMRA cells decreased (Philippe et al., 2019). Six weeks of a combined strength and endurance training increased the CD4+/CD8+ cell ratio in the elderly (Despeghel et al., 2021). However, further studies need to confirm these results as well as recommendations for the type, intensity, and duration of exercise.

### Exercise affects polarization of macrophages and systemic inflammation

Regular exercise also affects components of the innate immune system. Accordingly, it was shown in animal experiments that regular treadmill exercise induced the conversion of M1 to M2 macrophages, which corresponds to a change from a classical, more pro-inflammatory type to an alternative, more immunoregulatory type. This altered polarization is associated with a change in the expression profile of cytokines towards more anti-inflammatory messengers (Gleeson et al., 2011; Oliveira et al., 2013). An important mechanism shows that anti-inflammatory signals occur from the active skeletal muscle itself. Thus, during muscle contraction, increased IL-6 is released, described as myokine. IL-6 has an anti-inflammatory effect through exercise by stimulating the production of immune regulatory mediators such as IL-10 and the IL-1 receptor antagonist (Steensberg et al., 2003) as well as the downregulation of TNF-α by monocytes and macrophages (Starkie et al., 2003). IL-7 (Haugen et al., 2010) and IL-15 (Rinnov et al., 2014) are also myokines that may stimulate lymphocyte proliferation. IL-7 is assumed to exert a protective effect on the thymus. IL-15 appears to have effects on the induction of better survival of naïve T cells. Both cytokines were increased in subjects who were physically active throughout their lives, compared to their inactive controls (Duggal et al., 2018). In contrast to endurance training, which can reduce systemic IL-6 and TNF-α levels (Zheng et al., 2019), resistance training seems to have no or marginal effect on chronic inflammation (Rose et al., 2021). A combination of endurance and resistance exercise seems to affect basal levels of pro- or anti-inflammatory cytokines like IL-6, -8 and -10, even in the presence of inflammatory comorbidities (Despeghel et al., 2021). Similarly, it was also shown that exercise in postmenopausal obese women with dyslipidemia reduces systemic levels of TNF-α, which in turn has positive effects on metabolic health (Biteli et al., 2021).

## Exercise-effects on stress proteins

**FIGURE 1:**
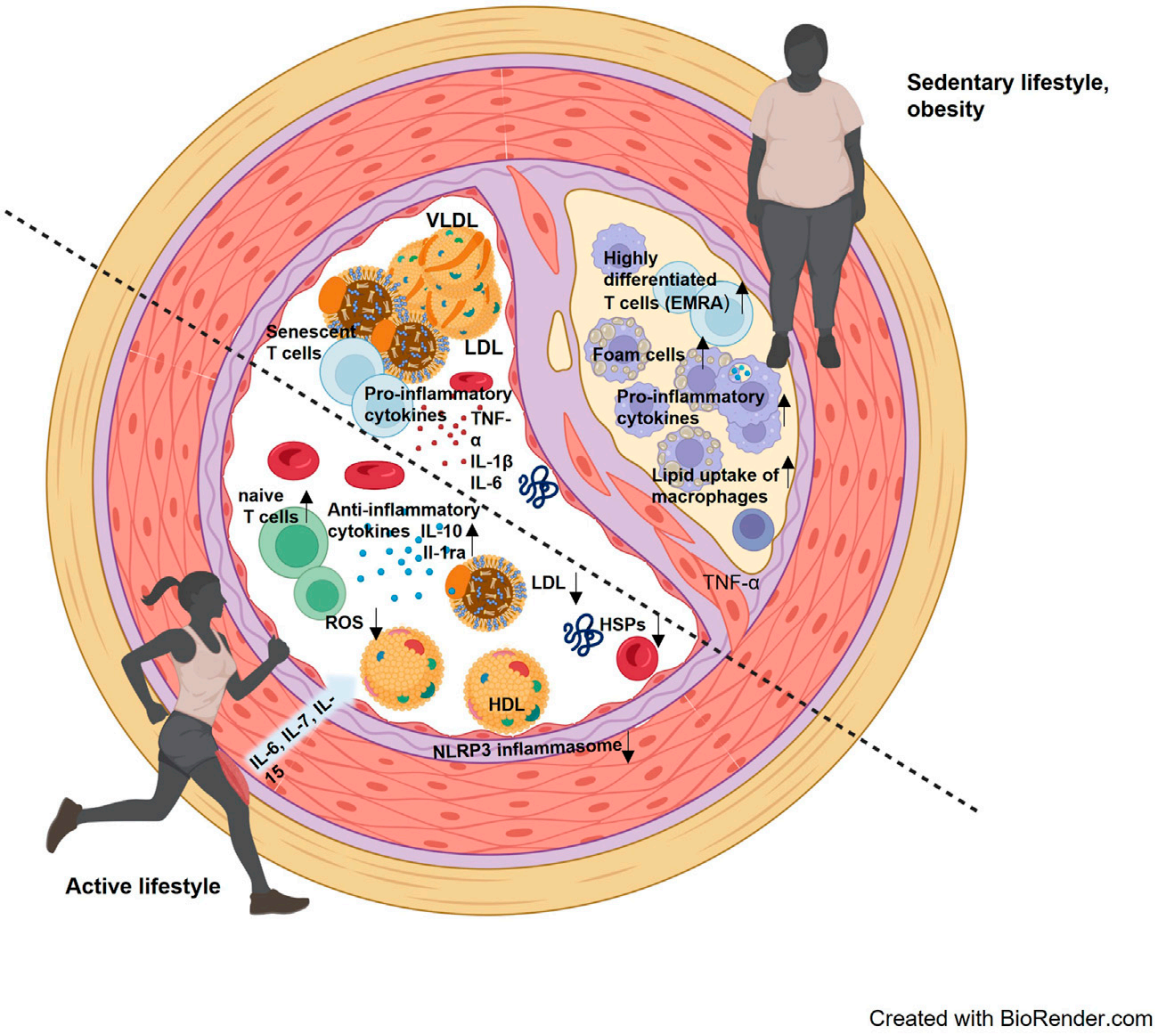
Interaction of blood lipids, cellular and molecular components of the immune system and stress proteins in an inactive lifestyle and an active lifestyle. The lower left half of the vessel visualizes the consequences of an active lifestyle, where reactive oxygen species (ROS) are effectively reduced, a more anti-inflammatory environment prevails, and blood lipids are present with a rather low LDL and higher HDL. The contrast is the upper right half of the vessel, where a more proinflammatory environment prevails, with higher LDL levels and accumulated senescent cells. An arteriosclerotic plaque has also already formed here, containing foam cells and infiltrated by more inflammatory leukocyte subpopulations.

Long-term exercise training improves the intracellular HSP response, which was demonstrated in muscle tissue and leukocytes (Fehrenbach et al., 2001; Yamada et al., 2008). Specifically, the expression of intracellular Hsp72 is suggested to be important to stabilize intracellular processes during conditions of increased oxidative stress which occurs in the context of systemic inflammation and dyslipidemia. (Morimoto, 1993; Febbraio and Koukoulas, 2000). Accordingly, we suggest that physical activity can strengthen cellular resilience by means of increased intracellular HSP induction, but at the same time prevent excessive release of HSPs into the extracellular space (Figure 1).

## Practical implementation of exercise training

According to the S1 Guideline “Screening in sports”, a sports medical screening should be carried out in the sense of a health examination to detect latent or pre-existing diseases that may pose a risk. Previous data indicate that targeted sporting activities have greater effects on changes in the lipid profile than a non-sport-based increase in everyday activity. Nevertheless, even more active daily life, which includes walking and strolling, climbing stairs and various physical activities of daily living, can contribute to the positive modification of plasma lipids. If we look at controlled exercise training, greater effects can be expected, especially through regular moderate and endurance activities. Current data recommend exercising for at least 30 min (or more) on as many days as possible. These amounts can also be started at a reduced level (e.g., shorter units of 10 min duration) and increased over time.

Practically, such activities can be achieved as walking and Nordic walking, jogging runs or cycling rides, by swimming or by participating in “cardio courses” in fitness facilities (Pressler, 2017). Intensities in the basic endurance range, i.e., below the individual anaerobic threshold, are recommended for such training units. Patients can be told as a rule of thumb for moderate-intensity aerobic training that you are active at a level that increases breathing and heart rate but still allows you to maintain yourself. In the area of endurance training, numerous health effects of High-Intensity Interval Training (HIIT) have also been demonstrated in recent years. This involves short-term intensive interval units that are usually performed on an ergometer or in running. At present, there is not enough data to assess the long-term effects of this type of training on a given lipometabolic disorder. Nevertheless, the studies show that HIIT also has significant effects on fat metabolism. Strong adrenergic innervation can improve the sensitivity of HSLs in adipose tissue, reduce metabolic stress in visceral fat and induce anti-inflammatory effects via stimulation of steroid hormone biosynthesis pathways (Sun et al., 2020). Accordingly, so-called “polarized training”, which is a combination of basic and HIIT training, can certainly be an effective training for dyslipidemia when individually designed. The effectiveness of pure strength training on the lipid profile is contradictory in the literature. Nevertheless, strength training is always considered useful in the context of general health promotion in order to provide repeated stimuli to maintain muscle mass and thus functionally maintain a certain “everyday athleticism”, prevent sarcopenia and promote the resilience of the musculoskeletal system (Barajas-Galindo et al., 2021). Here, the recommendations are 8–15 repetitions per exercise, which should be performed at least twice a week. This includes a 5–10 min warm-up with light aerobic activity. Each strengthening exercise should also be technically prepared so that it is performed correctly.

If statin therapy is indicated, it can be combined with activity and sports programs without any problems. If patients follow the activity recommendations in the long term, effects on blood lipids, especially on HDL and triglycerides, can already be expected after a few weeks. Finally, it should be mentioned that most patients with lipometabolic disorders have multiple morbidities that are influenced by physical activity. Here, it is particularly important to clarify the patients’ fitness for sports by means of a sports medicine stress examination, depending on the indication.
